# Esthetic Rehabilitation with Direct Composite Resin in a Patient with Amelogenesis Imperfecta: A 2-Year Follow-Up

**DOI:** 10.1155/2019/8407025

**Published:** 2019-08-14

**Authors:** Nathaly Stephania Palacios Rizzo, Leonardo Fernandes da Cunha, Bruno Vinueza Sotelo, Carla Castiglia Gonzaga, Gisele Maria Correr, Ubiracy Gaião

**Affiliations:** Graduate Program in Dentistry, Universidade Positivo, Curitiba, Brazil

## Abstract

Amelogenesis imperfecta is a group of conditions caused by over 15 different genes that affects the development of dental enamel and poses some challenges to dentists. An adult patient with amelogenesis imperfecta with severe changes in tooth color and reduction of occlusal vertical dimension sought dental treatment. Diagnostic wax-up was carried out to guide the stratification of a nanoparticulate resin for the restorative treatment. Direct composite resin restorations were applied on all teeth for modification of both esthetics and occlusion. After a 2-year follow-up, the findings appear to suggest that composite resin is a low-cost alternative when compared with indirect ceramic restorations, provides a good esthetic outcome, and offers considerable longevity for cases like the one reported herein.

## 1. Introduction

Amelogenesis imperfecta (AI) is a group of conditions caused by over 15 different genes that affects dentition, with variable prevalence rates depending on the population assessed. More often than not, AI patients have difficulty maintaining oral hygiene, impaired chewing ability, and lower self-esteem, which eventually affect their overall quality of life [1, 2]. There are numerous classifications based on phenotype. According to these phenotypes, AI can be categorized into type I that involves disturbances related to enamel secretion (hypoplastic), type II that is related to enamel maturation (hypomature), type III that affects the mineralization process (hypocalcified), and type IV, which is marked by the involvement of hypoplastic and hypomature enamel defects associated with taurodontism. In the hypoplastic forms, the enamel does not develop to its normal thickness; in the hypocalcified forms, the enamel thickness on newly erupted teeth closely approaches that of normal teeth, but the enamel is soft, friable, and can easily be removed from the dentin [1, 2].

Moreover, most AI cases require long-lasting and extensive dental treatment. Either indirect or direct restorations can be used for treatment. The approach should consider whether the enamel is sufficient for the restoration, in such a way that direct composite resin restoration can disguise the change in color and improve tooth morphology. In those AI patients in whom enamel is not sufficient for bonding, indirect restorations that completely cover the tooth structure are recommendable [1]. Clinicians should therefore think of treatment alternatives that could achieve the right balance between esthetics, patient's functional needs, and treatment costs.

Direct composite resin restorations can disguise tooth discoloration and improve dental esthetics, in addition to requiring less preparation or no preparation at all for preserving the tooth structure. This is therefore a good option for AI patients [3]. Currently, several resins are commercially available. Nanoparticulate resins allow adequate polishing and are resistant enough to maintain the esthetics and function of the guides used for disclusion and of restorations in case of extensive rehabilitations [4]. Moreover, they are less time-consuming and less costly, since they do not require any sessions for molding and testing, unlike indirect restorations.

The aim of the present study is to report a clinical case of an AI patient treated with direct composite resin and followed up for 2 years.

## 2. Case Presentation

**Figure 1:**
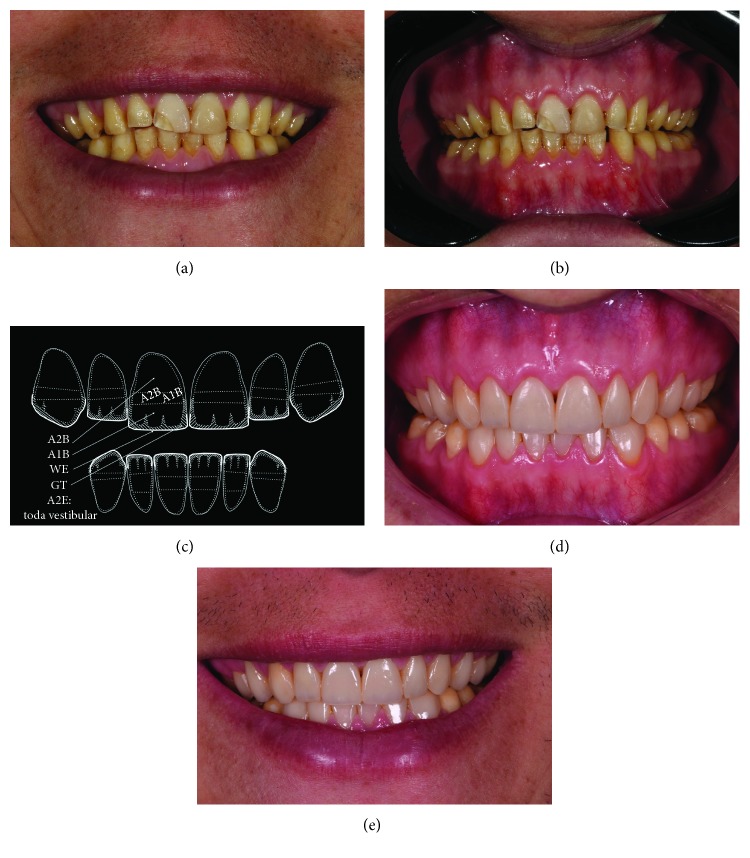
(a, b) Initial appearance of patient's teeth with amelogenesis imperfecta. (c) Selection of resin colors. (d, e) Restorations after occlusal adjustment and approximate view of restorations after final polishing.

A 38-year-old male patient sought dental treatment because of esthetic dissatisfaction with his smile. Amelogenesis imperfecta (AI) was clinically diagnosed (Figures 1(a) and 1(b)). The enamel was thin and was hypomineralized. Medical history was verified and his father and son also presented AI. The X-ray showed a healthy dental pulp. The patient was initially treated with prophylaxis and received oral hygiene instructions.

Thereafter, diagnostic wax-up was carried out. A direct restorative system was chosen. The colors for the dentin, enamel, and incisal edge were then selected.

The old and fractured veneers were removed. The operative field was totally isolated with a rubber dam and kept in position with an orthodontic elastic for gingival retraction in the cervical region. All the teeth were prepared with rotary instruments. The whole enamel surface of maxillary incisors was etched with 34% phosphoric acid to prevent application of the resin on the unetched area. The surface was then dried and Single Bond Universal (3M, USA) adhesive was applied with a regular-sized microbrush (Original Microbrush, Microbrush International). Polymerization was performed according to the manufacturer's instructions (Radii Plus SDI).

Stratification of the composite resin was initially performed on the palatal surface by applying a translucent GT resin. After that, a layer of A1B resin was used to create mamelons in the incisal region, leaving a space in the proximal region for allowing a more translucent area with the use of the GT resin. An A2B resin was applied at the middle third. The incisal halo was obtained with a WE resin. A single layer of A2E resin (Filtek Z350 XT, 3M) was applied on the whole buccal surface to provide a more esthetically pleasing contour (Figure 1(c)).

All resin increments were polymerized according to the length of time recommended by the manufacturer, in a continuous fashion and as close as possible to the resin, but without any contact with it, using a LED device (Radii Plus, SDI).

The same sequence was used for all anterior teeth. A mouth mirror was used to check for the presence of any defects between the dental surface and the resin on the buccal and palatal surfaces.

After taking the rubber dam out, excess resin was removed and the initial finish was obtained. Incisal edges were trimmed with carbon strips. After 24 h, finish and final polishing were obtained. Sandpaper strips were used in the proximal regions, and the contour of restorations was shaped using abrasive discs (Sof-Lex Pop-on, 3M), followed by the use of rubber cups (Jota do Brasil), felt wheels (American Burs) with composite resin polishing paste, and a brush impregnated with silicon carbide.

**Figure 2:**
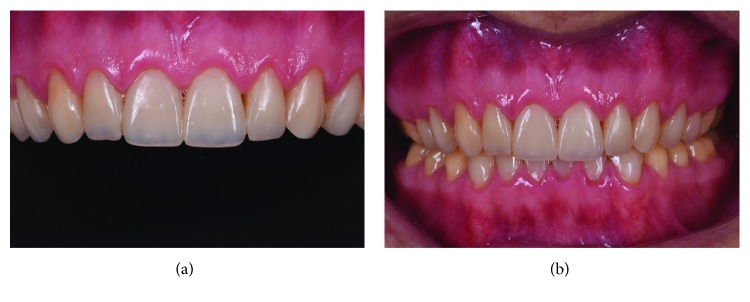
(a, b) 2-year follow-up.

The final appearance of restorations is shown in Figures 1(d) and 1(e). The patient returned for a follow-up visit after 2 years, as shown in Figure 2. No resin loss or fractures were observed after 2 years. However, staining was observed. Thus, polishing procedure was performed with discs, rubber cups, and felt wheels with polishing paste.

## 3. Discussion

AI affects mainly the quality and/or amount of dental enamel. Treatment plans of AI patients should consider patient age, socioeconomic background, and severity of the disorder [5]. Direct restoration was chosen in the present study because the patient had enough enamel for adequate bonding and also because of the lower cost of treatment as compared to indirect restorations. In addition, enamel deficiency makes teeth extremely sensitive to thermal and contact stimuli. Composite resin restorations are a solution to this problem as they protect the remaining structure. After restoration of all teeth with a direct composite resin, the patient no longer had esthetic complaints and he could restore his normal eating habits [5].

As the case reported herein was highly complex, a good-quality direct restorative system was used, allowing for a broad array of colors and multiple opacities, thus ensuring that the restorations could mimic the appearance of natural teeth. Because this system contains nanoparticles, its polishing provides high luster and its longevity in the oral cavity is increased [5, 6].

Initially, anterior teeth with any defects were waxed to obtain a more predictable increase of incisal edges [7]. Owing to stratification with different colors, the time required for restoration of a single tooth is longer. Therefore, several treatment sessions were necessary. Polishing of the restorations depends on the restorative and polishing materials used. Janus et al. observed good outcomes with the use of Filtek Z350, which contains nanoparticles only, when in association with Sof-Lex discs, as carried out in the present study [8].

## 4. Conclusion

AI is an inherited disorder that may be treated with direct composite resin restorations, thus improving esthetics and having long-term outcomes, performed quickly, safely, and efficiently.
